# Assessing Primary Neurogenesis in *Xenopus* Embryos Using Immunostaining

**DOI:** 10.3791/53949

**Published:** 2016-04-12

**Authors:** Siwei Zhang, Jingjing Li, Robert Lea, Enrique Amaya

**Affiliations:** ^1^The Healing Foundation Centre, Faculty of Life Sciences, University of Manchester; ^2^Department of Cell and Molecular Biology, Feinberg School of Medicine, Northwestern University; ^3^Department of Craniofacial Development and Stem Cell Biology, Dental Institute, King's College London

**Keywords:** Neuroscience, Issue 110, *Xenopus*, CNS development, immunostaining, neuronal differentiation, primary neurogenesis

## Abstract

Primary neurogenesis is a dynamic and complex process during embryonic development that sets up the initial layout of the central nervous system. During this process, a portion of neural stem cells undergo differentiation and give rise to the first populations of differentiated primary neurons within the nascent central nervous system. Several vertebrate model organisms have been used to explore the mechanisms of neural cell fate specification, patterning, and differentiation. Among these is the African clawed frog, *Xenopus*, which provides a powerful system for investigating the molecular and cellular mechanisms responsible for primary neurogenesis due to its rapid and accessible development and ease of embryological and molecular manipulations. Here, we present a convenient and rapid method to observe the different populations of neuronal cells within *Xenopus* central nervous system. Using antibody staining and immunofluorescence on sections of *Xenopus* embryos, we are able to observe the locations of neural stem cells and differentiated primary neurons during primary neurogenesis.

**Figure:**
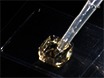


## Introduction

In vertebrates, the development of the central nervous system comprises several distinctive yet consecutive stages. The first step is neural induction, when naive ectodermal cells are specified toward a neural fate rather than an epidermal fate. Several interconnected regulatory mechanisms are involved during this stage in *Xenopus* and other model systems^ 1,2^. This process is primarily coordinated by secreted factors produced by the underlying mesoderm, such as chordin, noggin, and follistatin ^3-7^. After neural induction, a subset of neural progenitors exit the cell cycle and begin to differentiate in a process referred to as primary neurogenesis. Not all neuronal precursors differentiate at this time. The remaining neural precursor cells continue to proliferate, thereby maintaining the stem cell pool needed for continued growth of central nervous system throughout development and into adulthood.

These proliferating neural precursor cells are characterized by their expression of the *SRY (sex determining region Y)-box 3* (*sox3*) gene ^8-11^. The other population of cells, which exit the cell cycle and commit to a differentiated fate, are identified by the expression of the differentiation gene markers, *tubulin, beta 2B class IIb* (*tubb2b,N-tub*) and *myelin transcription factor 1* (*myt1*) *^12-14^*. Such differentiated neuronal cells eventually give rise to different categories of neurons including, but not limited to, motor, inter-, and sensory neurons positioned in distinct areas within the neural tube ^15-17^.

While significant efforts have been devoted to uncovering the regulatory mechanisms that govern the patterning and fate determining events in the anterior neuroectoderm, less attention has been made on investigating the neurogenic events that occur following the initial patterning stage. Indeed, signal transduction, transcriptional regulations, as well as post-translational modifications are all involved in this later stage, controlling both timing and lineage specification during neurogenesis^ 18-20^. Further investigations into these mechanisms require a reliable method to easily visualize and distinguish different populations of neuronal cells. The above-mentioned neural markers, including, Sox3, Myt1, and N-tub, can provide a means for identifying these different cell populations, thus providing the necessary foundations for revealing the underlying mechanisms of neuronal differentiation ^21-23^.

Although differential labeling of neuronal cell populations have been demonstrated in other model organisms, relatively few studies have exploited the *Xenopus *system to its fullest in this regard. This is mainly due to a paucity of compatible antibodies that reliably identify the various neuronal cell populations in the neural tube. Here, we describe a method for visualizing neuronal differentiation in early *Xenopus* embryos via immunostaining, which provides a robust and convenient approach for investigating primary neurogenesis in *Xenopus*. This protocol should give sufficient guidance for researchers interested in the early development of *Xenopus* central nervous system between stage 26 and stage 45.

## Protocol

All animal experiments were approved from the University of Manchester Animal Welfare Centre and were covered by a UK Home Office Project License.

### 1. Collection and Fixation of *Xenopus* Embryos

1. Prepare Reagents and Materials for Experiments.
  1. Prepare 10x Marc's Modified Ringers (MMR) by dissolving 56.5 g of NaCl in approximately 800 ml ultrapure water and adding stock solutions of 1 M KCl, 1 M MgSO_4_, 1 M CaCl_2_, and 1 M HEPES pH 7.4 to achieve a final concentration of 20 mM KCl, 10 mM MgSO_4_, 20 mM CaCl_2_, 50 mM HEPES. Adjust pH to 7.4 by 10 M NaOH and then adjust the final volume to 1 L.
  2. Sterilize the 10x MMR solution by autoclaving at 121 °C for 20 min on a liquid cycle. Upon using, dilute with ddH_2_O to 0.1x final concentration and add 20 mg/L gentamycin to inhibit microbial growth.
  3. Make 10x TBS solution by mixing 24 g Tris-HCl, 5.6 g Tris-base, 88 g NaCl and dissolving in approximately 900 ml ultrapure water. The final solution will have a pH value around 7.6. Adjust with either 10 M NaOH or concentrated HCl to achieve a final pH of 7.6 and final volume to 1 L.
  4. Upon using, make 1x TBS by diluting 1 part of 10x TBS solution with 9 parts of ultrapure water.
  5. Make 10x MEM Salt by dissolving 209.2 g MOPS in approximately 800 ml ultrapure water and adding stock solutions of 0.5 M EGTA and 1M MgSO_4_ to achieve a final concentration of 20 mM EGTA, 10 mM MgSO_4_. Adjust pH to 7.4 by 10 M NaOH and then adjust the final volume to 1 L.
  6. Sterilize the MEM salt solution by autoclaving at 121 °C for 20 min on a liquid cycle (the solution may turn yellow by a few months of storage at room temperature or after it has been autoclaved, but this change in color does not affect its use). However, do not use the solution after prolonged storage (more than 6 month).
  7. Make 1x MEMFA solution by diluting 1 part of MEM Salts, 1 part of 37% formaldehyde with 8 parts of Ultrapure water (v/v, stable at 4 °C for at least 1-2 weeks).
  8. Prepare 4% paraformaldehyde in TBS (for subsequent staining involving phalloidin) by dissolving 4 g of paraformaldehyde powder in 100 ml of 1x TBS solution Heat the solution to 60 °C and add a few drops of 10 M NaOH to assist dissolving. Aliquot in 5-10 ml volume and freeze in -20 °C. Do not re-freeze once thawed. CAUTION: Paraformaldehyde powder is an irritant and is toxic if inhaled, thus the weighing step should be performed in a fume hood.
  9. Label as many 4 ml glass vials with screw caps prior to sample collection.
  10. Prepare 15% gelatin/15% sucrose by pouring 20 ml of 40% fish gelatin (pre-heat in a 50 °C water bath) into a 50 ml centrifuge tube. Add 8 g of sucrose and fill the tube to the 50 ml line with 1x TBS.
  11. Place the gelatin tube on a rotary mixer or rolling bed to mix overnight at room temperature. This gelatin solution is stable at 4 °C for 1 week. Do not use expired solution and do not freeze-thaw.
2. Prepare and Fix *X. laevis* or *X. tropicalis* Embryos Cultured to Desired Stages.
  1. Culture the fertilized *X. laevis* or *X. tropicalis* embryos in 0.1x MMR with gentamycin until the desired stages. NOTE: Generally, collect embryos between stages 23 and 40. Later collection of embryos after stage 40 is possible, especially when observing axonal growth from the spinal cord, but keep in mind that additional gelatin penetration time may be required. The embryos may be wild-type, transgenic, mutant, inhibitor-treated, Morpholino (MO)-injected, or electroporated ^23,24^.
  2. Collect 20-50 embryos in each 4 ml glass vial, remove as much medium as possible and replace with MEMFA. NOTE: If subsequent staining involves phalloidin, use 4% paraformaldehyde instead of MEMFA since commercial supplied formaldehyde solutions usually contain up to 10% methanol as a stabilizer that will interfere with phalloidin staining.
  3. Fix overnight at 4 °C or, if in an urgency, at room temperature for 2 hr on a rotary mixer. The embryos will be stable in fixation solution for at least 1 week at 4 °C.
  4. After fixation, wash the embryos 3 times for 20 min using 1x TBS with 0.05% Triton X-100. After the final wash, remove as much TBS-Triton as possible and add 3 ml of 15% gelatin/15% sucrose into each vial.
  5. Place the vials on a roller bed overnight at room temperature. For embryos older than stage 40, use at least 24 hr of penetration time. After penetration, proceed immediately to section 2.2 on the next day.

### 2. Mounting and Cryosectioning of *Xenopus* Embryos

1. Prepare Reagents and Materials for Experiments.
  1. Take one box of positively charged slides, ideally unopened. NOTE: If opened, keep the slides in a dry condition (such as a dry box) and use within 1 month to ensure the static charge on the slides is maintained. Do not use expired slides since samples will fall off during immunostaining.
  2. Pre-chill the cryostat chamber to -30 °C. Set the instrument parameters as -35 °C for microtome and 12 µm section thickness. Install the thick cover glass plate onto the stage and let the cryostat equilibrate for at least 30 min before section starts.
  3. Prepare painting brushes for moving section strips, keep inside the cryostat chamber.
  4. Prepare pencils for writing on slides, put them at room temperature.
  5. Wear gloves during cryosection. Do not use bare hands.
2. Mounting *Xenopus* Embryos
  1. Carefully aspirate 5-10 embryos out of the glass vial using a plastic or glass pipette without introducing air bubbles. Transfer the embryos into the mounting chamber and observe under a stereoscope. Fill up the mounting chamber with gelatin solution to ensure the rigidity of the section block.
  2. Arrange the embryos as per **Figure 1** using a pair of fine-tip forceps. Mark the orientation of heads by drawing an arrow on the rim of the chamber using a cryogenic-compatible marker. For multiple groups of embryos, write down the description of each group on the rim of the corresponding chamber as well.
  3. Carefully place the chamber horizontally in a foam box half-filled with dry ice and close the lid. Observe the mounting chamber freeze in 5-10 min. Process each chamber serially (*i.e.* place the previous chamber onto dry ice before proceeding to the next one) as this will leave sufficient time for each chamber to freeze and prevent the dry ice box to become overcrowded. NOTE: Frozen mounting chambers do not need to stand horizontally and can be stacked up inside the box.
  4. Proceed with cryosectioning or, if required, keep frozen samples at -80 °C for at least 1-2 week without losing immunogenicity.
3. Cryosection of Mounted *Xenopus* Embryos
  1. Remove a frozen sample block from the chamber by pressing the bottom of the chamber using a blunt stick (such as a pencil).
  2. Add several drops of tissue-freezing medium, (a polyethylene glycol and polyvinyl alcohol-containing medium) onto the sample holding disc and mount the sample block with the anterior end of the embryos points up (**Figure 2**). Allow the mounted- block stand inside the cryostat chamber for approximately 1 min or until the tissue-freezing medium becomes opaque.
  3. Immediately install the sample holding disc onto the microtome with the bottom side of the sample block facing up. Trim off a portion of the sample block using a blade when the sample block is still relatively "soft" to reduce the length of each section (if desired). Leave the sample holding disc on the microtome for at least 5 min to allow its temperature to reach equilibrium.
  4. Gradually trim the sample block down until the heads of tadpoles are visible through the translucent gelatin. To increase trimming speed, apply a higher (thicker) setting of section thickness (*e.g.* 20-25 µM) at this stage (such as enabling the "trim" option) but not too thick because sample block can fall off from the sample holding disc. Observe the performance of cryostat, such as the sharpness and angle of the blade at this stage to ensure the generation of a long strip of subsequent sections.
  5. Once the tadpole heads become visible, adjust the settings of the cryostat back to normal (*e.g.* 10-12 µM) and clean both the microtome and stage using a paint brush. Gently make 2-3 sections as a trial using the hand wheel setting (do not use the motorized setting) to make sure that finished slices can form strips, not overlapping or sticking to the blade.
  6. Continue trimming until the heads of tadpoles are almost exposed (this may need some experience but achievable). Brush off any remnant sections on the stage and the collection of sample sections will begin.
  7. Make approximately 10-15 sections and let them form a long strip.
  8. Flip over the thick cover glass plate to the side and gently remove the strip from the blade using a fine-tip paint brush and arrange it on the stage with long axis parallel to the blade.
  9. Pick one positively charged slide at room temperature, label it with a pencil (which will not be washed off during subsequent staining treatments), press it quickly but firmly onto the strip with the label side facing down and remove the slide from the cryostat chamber. If done correctly, the strip should immediately stick to the positively charged slide (**Figure 3A**).
  10. Repeat this step to have 20-30 slices on each slide and arranged in parallel (**Figure 3B**). This is sufficient to cover the entire brain region of *X. tropicalis* embryos and most of forebrain and midbrain regions of *X. laevis* embryos.
  11. Air-dry the slides for 10 min, proceed immediately or, store the slides in a slide box at -80 °C for at least 3-6 months without losing immunogenicity.

### 3. Immunostaining of Sectioned *Xenopus* Embryos

1. Prepare Reagents and Materials for Experiments.
  1. Prepare 0.05% TBS-Triton X-100 by adding Triton X-100 into 1x TBS to achieve a final concentration of 0.05%. This solution is stable at room temp for 3-4 days. Do not use expired solution.
  2. Prepare 5% heat-inactivated goat serum or 5% BSA in TBST as blocking buffer. Firstly, heat-inactivate the goat/lamb serum by placing approximately 20-30 ml of serum in a 65 °C water bath for 30-60 min. Aliquot into 1.5 ml centrifuge tubes and freeze in -20 °C. No re-inactivation is required upon using.
  3. Dilute the heat-inactivated serum or BSA in TBST to achieve a final concentration of 5% before use.
  4. Prepare appropriate primary antibodies according to dilutions in blocking buffer (*e.g. *1:500 anti-Sox3 ^10,25^/1:250 anti-Myt1 ^26^/anti-acetylated tubulin). Use approximately 100 µl of antibody solution on each slide.
  5. Prepare appropriate fluorescent antibodies (*e.g.* anti-mouse/rabbit red fluorescent dye-conjugated antibodies) in blocking buffer (usually 1:500).
  6. (optional) Add red fluorescent dye-conjugated Phalloidin (1:500) into secondary antibody mix to reveal actin network.
  7. (optional) Add DAPI (0.5 µg/ml final concentration) into secondary antibody mix to visualize nuclear localization.
  8. Take the anti-fade mounting medium out of the freezer and thaw in room-temperature water bath.
2. Immunostaining
  1. Remove the frozen slides from -80 °C and place them on a piece of towel paper inside a ventilation hood for at least 1 hr to eliminate any condensed water droplets, then bake the dried slides on a 85-90 °C heat block with the slices facing up for 15 min to activate the adhesion mechanism. Finally, allow the slides cool down to room temperature (at least 10 min).
  2. Fill the staining jar with pure acetone and incubate the slides in acetone for 10 min to remove fish gelatin. If multiple slides are to be treated, arrange them on a staining rack. Air-dry the treated-slides for 15 min in a ventilation hood. Do not re-use the acetone.
  3. Carefully draw a ring around the samples on the slide using a PAP pen without touching the samples. Ensure that the ring is self-enclosed, otherwise antibody solution will flow out during staining. Fully dry the PAP pen ring.
  4. Fill another staining jar with 1x TBS without Triton X-100. Insert the dried-slides into the staining jar and allow rehydration for at least 1 hr. In the meantime, prepare the blocking buffer by diluting either heat-inactivated goat serum or BSA to 5% final concentration using 1x TBS with 0.05% Triton X-100.
  5. Make a wet box by placing 1-2 6-well plates inside a click-lock food box and fill the wells halfway with ultrapure water. Remove rehydrated slides from the staining jar and place them horizontally on the 6-well plates (without the plate lid).
  6. Carefully add 300-600 µl of blocking buffer inside the PAP ring. Seal the wet box by locking the lid into position and incubate at room temperature for at least 1 hr.
  7. Dilute the appropriate primary antibodies in blocking buffer. Generally, use 100-150 µl diluted antibody solution per slide. If multiple slides are processed, scale up the volume proportionally.
  8. After blocking, carefully remove the blocking buffer from slides by aspiration and quickly add primary antibody solution to prevent dry-out, then seal the wet box and incubate at 4 °C overnight.
  9. On the next day, remove the primary antibody solution from the slides by aspiration. Wash the slides by inserting into staining jars filled with 1x TBS with 0.05% Triton X-100, 3 times, 15 min each. Meanwhile, dilute the appropriate fluorescent secondary antibodies with blocking buffer (with or without DAPI or phalloidin).
  10. Carefully add 100-150 µl of secondary antibody solution onto the slides. Place the slides inside the wet box and seal the lid. Incubate the slides for 1-2 hr at room temperature.
  11. Wash the slides in staining jars filled with 1x TBS with 0.05% Triton X-100, 3 times, 15 min each. At the final wash, thaw the anti-fade mounting medium in a 50 °C water bath for 10 min if stored at -20 °C. Cool down the mounting medium to room temperature before use.
  12. Add approximately 20 µl (one drop) of mounting medium onto the slide and apply a large cover slip (at least 22 mm x 64 mm) over the samples. Observe using fluorescent or confocal microscope within 6 hr post mounting.
    1. If imaging cannot be done on the same day, seal slides using nail polish and place them inside the wet box at 4 °C overnight. NOTE: The anti-fade mounting medium will gradually get oxidized after a few days (and turn brownish) so it is recommended to take the images as soon as possible.

## Representative Results

The representative results show cross-sections of stage 30 *Xenopus* embryos at different levels, namely the forebrain, midbrain, hindbrain, and spinal cord, stained with different antibodies (**Figure 4**). As mentioned, Sox3-labeled neuronal progenitor cell population locates in the proximity of the lumen of the neural tube, while MyT1-labeled differentiated primary neurons migrate outward and locate near the marginal layer (basal lamina) of the neural tube. Anti-acetyl-tubulin labels axons in differentiated neurons, which can be observed within the whole neural tube.

Side-specific gene knockdown or overexpression is a broadly used method in neurobiology to evaluate whether modulating the expression of specific gene(s) disrupts the growth and differentiation of neuronal cells. In such cases, either Morpholinos (MOs) or DNA construct(s) carrying promoter-driven gene(s) are injected into one of the two blastomeres at two-cell stage ^23^. Alternatively, they can be injected into the brain ventricle followed by electroporation, which will result in knockdown or over-expression of desired gene(s) ^27^. In both cases, the effects will be restricted to one side of the embryo, making the opposite side of the embryo as untreated internal control.

After fixation, sectioning, and immunostaining, the impacts of gene knockdown/over-expression are quantified by counting and comparing the cell numbers of different populations at both sides of embryo. By accumulating these data from several embryos, statistical analysis can be performed. In our representative results, no perturbation was made in the embryos. Examples of gene perturbation and their effects on different neuronal cell populations can be found in the references ^20,23^.


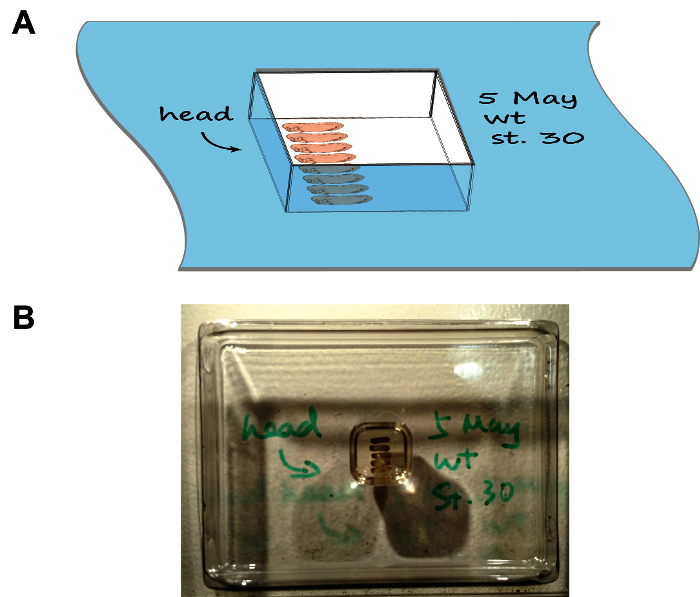
**Figure 1: Embryo arrangement and orientation in the section mould.** (**A**) A cartoon depiction showing the mounting assembly, note the tray has been labelled with the date, stage, and the orientation of embryos. (**B**) An image showing the natural appearance of the mounting assembly. Please click here to view a larger version of this figure.


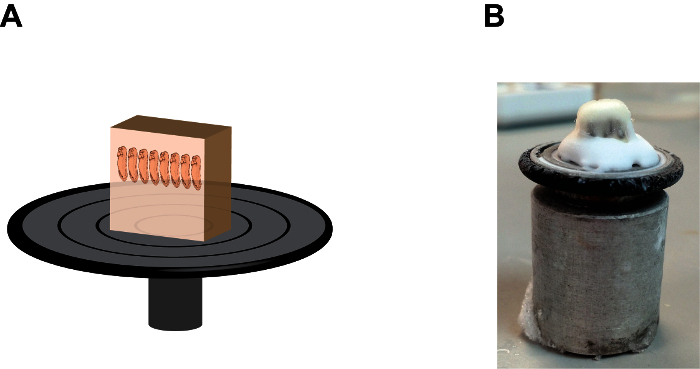
**Figure 2: The orientation and position of the gelatin block on the sample holding disc.** (**A**) A cartoon depiction showing the section block assembly, note that embryos are in a "heads up" position and the gelatin block is firmly fixed onto the sample holding disc by the tissue-freezing medium at the base (see Figure 2B). (**B**) An image showing the natural appearance of the section block assembly, note the accumulation of the tissue-freezing medium between the gelatin block and the sample holding disc. Please click here to view a larger version of this figure.


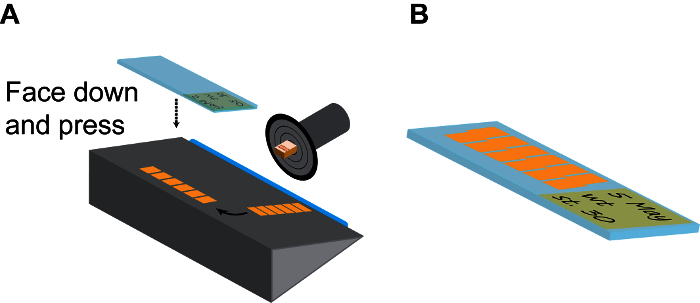
**Figure 3: Continuous sectioning.** (**A**) Model of a section chamber. The sample holding disc should be rotated and fixed to the position that the embryo side is facing up. After a few (6-10) sections, the long strip of continuous section tiles are gently separated from the blade (blue) using a fine brush, and then turned 90° on the holding plate. Then a room-temp positively charged slide is firmly pressed onto the section strip facing down and immediately lifted up to collected finished strips. (**B**) Normally, each slide can accommodate 2 parallel lines of strips, as shown. Remember to make detailed record on the slide label using pencil. Please click here to view a larger version of this figure.


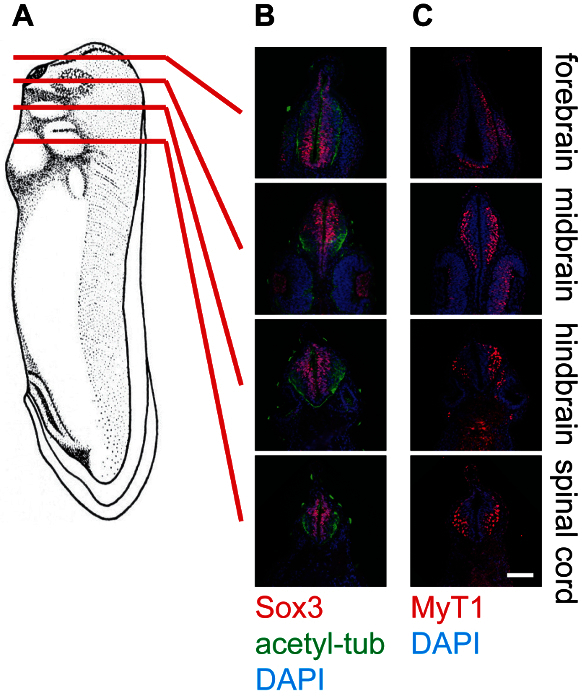
**Figure 4: Cross-section of stage 30 *****X. laevis***** tadpoles and staining in the neural tissue,20X. **(**A**) Schematics indicating the relative positions (forebrain, midbrain, hindbrain, and spinal cord) of section planes on *Xenopus* tadpoles. (**B**) Corresponding cross-sections stained with anti-Sox3 (Red), anti-acetylated-tubulin (Green), and DAPI (Blue), showing the relative locations of neural stem cell pools as well as neurofilaments within the neural tube. (**C**) Corresponding cross-sections stained with anti-MyT1 (Red) and DAPI (Blue), showing the relative locations of differentiated primary neurons within the neural tube. Scale bar = 20 µm. Please click here to view a larger version of this figure.

## Discussion

Here we demonstrate a convenient and efficient method for visualizing primary neurogenesis in *Xenopus* embryos. This method permits the assessment of different types of neural cells, including neuronal stem cells and differentiated primary neurons using cell type-specific markers.

The protocol is generally robust with very high level of reproducibility. For embryos that are at pre-hatching stages (*i.e. *up to stage 28), we recommend manually removing the vitelline membrane prior to fixation as this allows embryos to fully uncurl before fixation. This is especially important when collecting embryos at relatively early stages (before hatching), since bent embryos are extremely difficult to arrange at the desired orientation inside the mounting chamber. We have not experienced a loss of immunogenicity after MEMFA or PFA fixation hence additional antigen retrieval step is not necessary for this protocol. In addition, this immunostaining procedure can be carried out in samples from chromogenic/fluorescent in situ hybridization. In such scenario, it is recommended to skip the protease K treatment step during the in situ hybridization protocol. After chromogenic/fluorescent substrate reaction, samples can be washed with TBS and embedded in fish gelatin solution in a similar way to MEMFA/PFA fixed samples. Sample vials can be protected from light by wrapping the vials in aluminum foil if fluorescent in situ hybridization will be imaged together with immunofluorescent staining later.

In some cases, embryos may shrink excessively after gelatin penetration. This is most likely caused by either insufficient fixation or insufficient TBS-Triton extraction. Ensure that embryos have been fixed in PFA/MEMFA for at least 2-3 hr or preferably overnight at 4 °C. If the problem persists, increase the washing time of TBS-Triton to 3 for 1 hr.

During cryo-sectioning, the rigidity of sample block may vary as different batches of fish gelatin have different properties. This problem can be partially compensated by adjusting the temperature of the microtome. A lower temperature will result in a "harder" sample block however under excessive low temperature the sample block becomes crisp and difficult to section. Some fine-tuning or practice (using mock sample blocks without embryos) before each sectioning may be beneficial prior to use on particularly valuable samples.

A commonly encountered problem during sectioning is the thin sections sometimes tend to stick onto the thick glass plate rather than stay flat on the steel stage. This can be particularly disrupting since it constantly interrupts the continuous sectioning process. Such cases are usually caused by one of two reasons: the section chamber and (especially) the thick glass plate not being cold enough, or excessive static charge on the machine and the operator, especially in dry weather. The former can be solved by turning down the temperature of the section chamber and leaving the glass plate within for extra time (possibly overnight); and the latter by properly grounding the cryostat. Connecting the metal surface of the cryostat to a metal tap or similar water tubing system made of metal can be an alternative way to release the static charges. After each use, the thick glass plate should be washed well with non-corrosive detergent (such as dishwashing liquid), rinsed by ultrapure water, sprayed with pure ethanol, and wrapped in towel paper to protect the surface and edges from damaging.

The antibodies listed in the protocol are generally specific and we rarely encounter nonspecific adsorption or high background signal. It should be noted that, if using heat-inactivated goat/lamb serum as blocking agent, excessive heat-inactivation (as visualized by the formation of fluffy precipitant in the serum) should be avoided as this precipitant is highly attractive to secondary antibodies and may contribute to a source of high background.

It is possible, and sometimes desired, to perform double-staining to visualize different populations of neuronal cells simultaneously. Such double-staining is possible and generally gives satisfactory results with the following combinations: Sox3/N-tub or Myt1/N-tub. However, double-staining of Sox3 and Myt1 is complicated by the fact that the two antibodies are both from rabbit origin. We have tested several antibody direct labeling kits, however no satisfactory results were observed (low signal-to-noise level), possibly due to the lack of signal amplification from secondary antibodies. One possible approach to circumvent this problem would be to raise transgenic lines in *Xenopus*, as discussed below.

One of the main restrictions of this protocol is that, while this protocol could sufficiently distinguish two main pools of neuronal cells, namely, the Sox3-expressing neural stem cell pool and the Myt1-expressing differentiated neurons, it lacks the ability to reveal different sub-populations of differentiated neurons. Such sub-populations, which including, but are not limited to, primary motor neurons, interneurons, and sensory neurons, are usually characterized by their differentially-expressed marker genes ^28-30^. As mentioned above, recent advances in the development of multicolor *in situ* hybridization combined with antibody-based immunofluorescence detection method in *Xenopus* embryos may fill in the gap to reveal these sub-populations of differentiated neurons for potential investigation ^31^. Alternatively, as has been demonstrated in mice model, it would also be desired to raise a more comprehensive set of cell type-specific antibodies to distinguish such different cell populations in *Xenopus*.

It is also worth mentioning that, as an addition to this method, recent advances in optical imaging and image analysis, such as multiphoton microscopy, 3D reconstruction, and segmentation, can also be applied after initial assessments to achieve more comprehensive observations of *Xenopus* oocytes as well as early embryos, particularly under a live setting ^32,33^. Therefore, to track proliferation, differentiation, and movement of neuronal cells in live animals, it would be desired to establish one or more transgenic lines that harbor fluorescent proteins driven by cell type-specific promoter to allow live observation of such cell populations in early *Xenopus* embryos. The establishment of a *X. laevis* line with neuro-specific β-tubulin promoter driving tauGFP and its applications have provided a nice example ^23,34^. With the full promoter sequences of both *sox3* and *myt1* characterized in vertebrates ^35-37^, it is should be relatively easy to establish additional transgenic lines in *Xenopus* which should contribute extensively to both the *Xenopus* community and a more general field of primary neurogenesis research.

## Disclosures

The authors have nothing to disclose.
